# An Overview of Mitochondrial Protein Defects in Neuromuscular Diseases

**DOI:** 10.3390/biom11111633

**Published:** 2021-11-04

**Authors:** Federica Marra, Paola Lunetti, Rosita Curcio, Francesco Massimo Lasorsa, Loredana Capobianco, Vito Porcelli, Vincenza Dolce, Giuseppe Fiermonte, Pasquale Scarcia

**Affiliations:** 1 Department of Pharmacy, Health, and Nutritional Sciences, University of Calabria, 87036 Arcavacata di Rende, Italy; federica.marra@unical.it (F.M.); rosita.curcio@unical.it (R.C.); vincenza.dolce@unical.it (V.D.); 2 Department of Biological and Environmental Sciences and Technologies, University of Salento, 73100 Lecce, Italy; paola.lunetti@unisalento.it (P.L.); loredana.capobianco@unisalento.it (L.C.); 3 Laboratory of Biochemistry and Molecular Biology, Department of Biosciences, Biotechnologies and Biopharmaceutics, University of Bari Aldo Moro, via E. Orabona 4, 70125 Bari, Italy; francesco.lasorsa@uniba.it (F.M.L.); vito.porcelli@uniba.it (V.P.); 4 Institute of Biomembranes, Bioenergetics and Molecular Biotechnologies, National Research Council, 00155 Rome, Italy

**Keywords:** neuromuscular diseases, mitochondrial metabolism, OXPHOS, mitochondrial carrier family, neuromuscular junction, myopathy, MELAS, MERF, Leigh syndrome, therapy

## Abstract

Neuromuscular diseases (NMDs) are dysfunctions that involve skeletal muscle and cause incorrect communication between the nerves and muscles. The specific causes of NMDs are not well known, but most of them are caused by genetic mutations. NMDs are generally progressive and entail muscle weakness and fatigue. Muscular impairments can differ in onset, severity, prognosis, and phenotype. A multitude of possible injury sites can make diagnosis of NMDs difficult. Mitochondria are crucial for cellular homeostasis and are involved in various metabolic pathways; for this reason, their dysfunction can lead to the development of different pathologies, including NMDs. Most NMDs due to mitochondrial dysfunction have been associated with mutations of genes involved in mitochondrial biogenesis and metabolism. This review is focused on some mitochondrial routes such as the TCA cycle, OXPHOS, and β-oxidation, recently found to be altered in NMDs. Particular attention is given to the alterations found in some genes encoding mitochondrial carriers, proteins of the inner mitochondrial membrane able to exchange metabolites between mitochondria and the cytosol. Briefly, we discuss possible strategies used to diagnose NMDs and therapies able to promote patient outcome.

## 1. Introduction

Neuromuscular diseases (NMDs) include a group of genetically determined or acquired diseases that can involve different components of the motor unit and lead to the development of incorrect signals between motor neurons and muscle or, as occurs in most cases, a total loss of communication between these elements [1].

These degenerative pathologies can occur both during development and in adulthood. In particular, early onset (i.e., developmental) types are mostly genetically determined and are typically very severe, while those developing in adulthood include more inflammatory forms or are secondary to other pathologies [2]. Symptoms of neuromuscular diseases are a homogeneous group of disorders occurring at any time in life. The most frequent symptoms are muscle weakness, twitching, torpidity, cramps, rigidity, myalgia, and loss of muscular control, whose frequency and intensity can vary according to the extent, site, and trend over time in relation to the type of pathology [3]. In advanced stages, spasticity and total paralysis of the subject may occur. Furthermore, some forms of muscular diseases can involve respiratory and cardiac muscles, resulting in their impaired function and causing the premature death of the patient [4].

To date, the specific cause of all neuromuscular diseases is not well known, but research has shown that genetic mutations underlie most of them; in fact, more than 500 genes have been associated with the development of neuromuscular disorders. Such heterogeneity and complexity of genetic defects can make it difficult to find effective therapies suitable for different types of neuromuscular pathologies [5]. Hence, an accurate diagnosis is necessary to identify the type of neuromuscular disease and to develop targeted therapies for individual patients, thus improving their quality of life and mobility, as well as disease outcome.

Neuromuscular diseases include a very large group of disorders that differ from a genetic and pathological point of view. However, most neuromuscular diseases entail mitochondrial dysfunction [6]. In fact, several studies have shown that in most neuromuscular diseases, mitochondria are dysfunctional, thus causing alterations in the respiratory chain and energy production. This contributes to the development of pathological neuromuscular symptoms affecting both striated muscles and the nervous system.

The purpose of this review is to analyze the role of mitochondria in the onset of neuromuscular diseases by evaluating the involved proteins and their associated mitochondrial functions that appear to be altered.

## 2. Overview of NMDs’ Clinical Features

Neuromuscular diseases can be divided into two large groups: muscle and nerve pathologies. The former are characterized by a direct loss of muscle function, while the latter are related to an indirect loss of function of the nerves, which in turn affects muscle function [7]. Muscle pathologies include muscular dystrophies (MD) and myopathies, whereas neuromuscular junction (NMJ) disease and motor neuron disease are among the nervous ones [8].

Muscular dystrophies represent a heterogeneous group of skeletal muscle disorders involving the structure of muscle cells and result in their degradation. They include congenital muscular, Duchenne, facioscapulohumeral muscular, and myotonic dystrophies. Congenital muscular dystrophies are hereditary disorders characterized by a progressive clinical course resulting in muscle weakness and slow degradation of the joints with consequent deformity [9,10]. Duchenne dystrophy is a disease leading to muscle deterioration due to the lack of production of the dystrophin protein. It manifests from birth, causing motor impairment in a short time, with subsequent death from compromised respiratory muscles [11]. Facioscapulohumeral muscular dystrophy (FSHD) is the most common dystrophy, and its symptoms mostly involve the muscles of the arms, trunk, and face. FSHD is an autosomal dominant disease, genetically linked to deletions found in the chromosome 4q35 region [12,13], leading to an increase of oxidative stress and a decrease of the GSH/GSSG ratio, resulting in the impairment of mitochondrial energy production [14]. Myotonic dystrophies are characterized by the inability to relax muscles. This condition initially involves facial muscles but can also affect those of the hands, feet, and neck. In particular, myotonic dystrophy is distinguished as type 1 or type 2. Both forms are caused by autosomal dominant nucleotide repeat. In type 1, an unstable trinucleotide sequence, cytosine–thymine–guanine (CTG), repeat is present in the 3′UTR of the myotonic dystrophy protein kinase (DMPK) gene, encoding a myosin kinase expressed in skeletal muscles, whereas in type 2 an elevated repetition of four unstable nucleotides, cytosine–cytosine–thymine–guanine (CCTG), in the intron 1 of the cellular nucleic acid-binding protein (CNBP) gene (previously known as zinc finger 9 gene), encoding a protein able to bind DNA or RNA [15,16].

Myopathies are pathologies that affect the contraction of voluntary muscles and sometimes are secondary to muscle inflammation. They include congenital and inflammatory myopathies. In the latter case, mitochondrial dysfunctions and reactive oxygen species (ROS) level trigger muscular inflammation [6]. Congenital myopathies are a heterogeneous group of hereditary disorders of the skeletal muscles, damaging their tone and contractile capacity. The most frequent forms are called core myopathies and occur mainly during childhood, causing weakness of the limbs and facial muscles, hypotonia, and areflexia. They rarely affect adults, which experience muscle cramps and limb–girdle weakness [17,18]. Inflammatory myopathies, also called myositis, are heterogeneous pathologies characterized by muscle inflammation, and are often accompanied by extra muscular disorders affecting the skin, lungs, and joints. A particular form of inflammatory myopathy is called inclusion body myositis (IBM), and is characterized by an accumulation of degraded proteins in muscle fibers, which cause pain in the muscles of the lower limbs with difficulty in getting up [19,20].

NMJ diseases entail the destruction, dysfunction, or absence of one or several key proteins responsible for the transmission of signals between the nerves and muscles. In fact, the neuromuscular junction is an excitatory cholinergic synapse that releases acetylcholine (ACh) from the motor neuron terminal to the muscle fiber, generating an action potential responsible for the initiation of the excitation–contraction process [21,22]. Impairment of this synapse leads to the development of pathologies such as myasthenia gravis, Lambert–Eaton myasthenic syndrome, and congenital myasthenic syndromes. Myasthenia gravis is one of the most common forms of NMJ disease. It is characterized by the production of autoantibodies directed against various proteins at the level of the postsynaptic membrane. [23]. Only a small group of muscles is affected, including the extra ocular and bulbar muscles, as well as those of the upper limbs. In some cases, muscle weakness associated with myasthenia gravis can lead to the onset of respiratory infections and osteoporosis, with all the associated complications [24]. Lambert–Eaton myasthenic syndrome is an autoimmune neuromuscular disease characterized by the production of autoantibodies directed against voltage-gated calcium channels located at the presynaptic terminal. Alterations of these channels determine a reduced release of Ach [25], initially causing weakness in the legs and later in the arms. Ocular and bulbar dysfunctions can also occur with the evolution of the disease [26,27]. Congenital myasthenic syndromes comprise a group of approximately 30 pathologies. They develop as a result of an altered protein function occurring at the level of the presynaptic or post-synaptic membrane, as well as in the synaptic space. Most of them are characterized by an ACh receptor mutation [28]. Congenital myasthenic syndromes can occur from birth or in adulthood; in the first case, muscle weakness mainly involves the ocular and cranial muscles, whereas in the second case the proximal and trunk muscles are involved [29].

Motor neuron diseases are characterized by the progressive loss of function of lower motor neurons in the ventral horn of the spinal cord. Such pathologies affect both adults and children; in any case, symptoms are characterized by movement disorders and muscle weakness [30]. Motor neuron diseases include amyotrophic lateral sclerosis (ALS), spinal-bulbar muscular atrophy (SBMA), and spinal muscular atrophy (SMA) [31,32,33,34,35].

## 3. Role of Mitochondria in Neuromuscular Diseases

Neuromuscular diseases include a very wide range of conditions, making them difficult to diagnose and treat. Despite the broad heterogeneity, an increasing body of evidence indicates that mitochondrial impairment and dysfunction play an important role in the pathogenesis of such disorders, being the main abnormalities and prominent early features found in all NMDs [6,36,37].

Mitochondria generate, through oxidative phosphorylation (OXPHOS), most of the energy supply needed to fuel cellular activity. In addition, they regulate crucial cellular processes, including cell metabolism, calcium homeostasis, and programmed cell death. They are also the major source of ROS, which can influence homeostatic signaling pathways, as well as control cell differentiation and proliferation [38].

Mitochondria form a physically and functionally highly interconnected network in skeletal muscles and nervous tissues. This network undergoes constant changes in mitochondrial shape, size, and number in response to a variety of physiological and pathophysiological stimuli in order to meet cell energy demands [39,40,41,42,43]. Muscle and nerve cells have a high density of mitochondria being highly dependent on OXPHOS for energy production [44], thus explaining why they are extremely sensitive to energy-dependent defects. Indeed, mitochondrial impairment promotes the onset and progressive unfolding of several pathological conditions in humans, including neurodegenerative and neuromuscular disorders [45].

The central role of mitochondria in skeletal muscle and neuronal functionality depends not only on energy supply but also on the coordination of key processes defined as mitochondria quality control pathways, which preserve mitochondrial integrity and function [39,46], such as energy production, metabolism, intracellular signaling, and apoptosis. These pathways are essential for skeletal muscle in response to exercise and diseases [47]. 

Most neuromuscular syndromes due to mitochondrial dysfunction have been associated with a variety of biochemical and genetic defects. NMDs can be caused by mutations in either mitochondrial DNA (mtDNA) or nuclear DNA (nDNA) affecting genes involved in mitochondrial metabolism and bioenergetics [48]. Moreover, different mutations have been described in nuclear and mitochondrial genes encoding some subunits of the respiratory chain complexes and other mitochondrial enzymes, such as those involved in β-oxidation and the tricarboxylic acid cycle, as well as translocases, that can be implicated in various neuromuscular disorders [49].

In addition to genetic mutations, neuromuscular disorders often display reduced mitochondrial functionality in skeletal muscle, which is not directly related to mtDNA or nDNA mutations but is secondary to other diseases and conditions. In these cases, the propagation of mitochondrial dysfunction often occurs because of the imbalance between ROS generation and their detoxification [50]. Owing to their contractile activity, high oxygen consumption, and energy demand, skeletal muscles continuously produce moderate levels of ROS, which have essential functions in cellular signaling, by regulating the expression/activity of numerous genes and enzymes. However, the type of effect exerted by ROS is highly dependent on cellular antioxidant systems [6,51,52,53].

## 4. Alteration of the Respiratory Chain Enzymes in NMDs

Mitochondrial OXPHOS is the process producing the ATP needed by eukaryotic cells. Enzymatic reactions are composed by the electron transport chain and ATP synthesis machinery. The former consists of four multi-enzymatic complexes, which are located at the level of the inner mitochondrial membrane, and two mobile electron carriers, coenzyme Q and cytochrome c. Through these complexes, electrons are transferred from NADH and succinate to molecular oxygen; moreover, protons are released in the inner membrane space, thus generating a proton gradient that is indispensable for ATP synthesis by the F1Fo ATP synthase complex starting from ADP and inorganic phosphate [54,55]. The redox reactions are carried out by the following complexes: Complex I or NADH: ubiquinone oxidoreductase, Complex II or succinate dehydrogenase, Complex III or ubiquinol: cytochrome c oxidoreductase, and Complex IV or cytochrome c oxidase. Each of them is associated with different cofactors, including NAD, flavins, iron-sulfur clusters, hemes, and ions [56,57].

NADH: ubiquinone oxidoreductase represents the entry point of electrons into the respiratory chain. It transfers electrons entirely to ubiquinone [58]. This process is coupled with the translocation of four protons across the inner mitochondrial membrane. Furthermore, Complex I is one of the main sources of ROS production, which cause oxidative stress [59].

Succinate dehydrogenase participates both as a component of the electron transport chain and as an intermediate of the Krebs cycle, i.e., this enzyme catalyzes the oxidation of succinate into fumarate and, at the same time, it reduces ubiquinone to ubiquinol [60]. Succinate dehydrogenase is the only complex of the respiratory chain that does not pump protons through the inner mitochondrial membrane.

Ubiquinol: cytochrome c oxidoreductase transfers electrons from ubiquinone to cytochrome c with the translocation of four electrons across the mitochondrial membrane [61]. It also significantly contributes to ROS generation, and unlike Complex I that generates ROS only at the level of the mitochondrial matrix, Complex III releases ROS also into the intermembrane space [62].

Cytochrome c oxidase (COX) is the last enzyme of the electron transport chain and transfers electrons directly to molecular oxygen, leading to the formation of H_2_O, while two protons are pumped in the mitochondrial intermembrane space, thus increasing the transmembrane difference of proton electrochemical potential [63].

**Figure 1:**
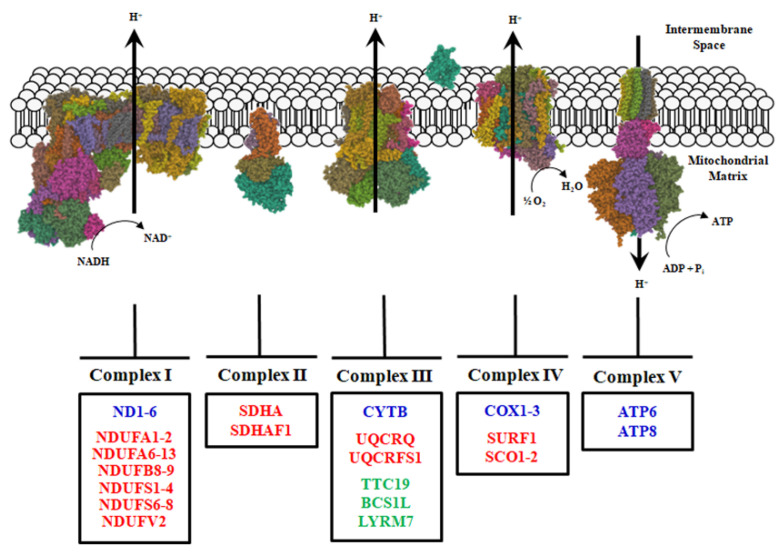
Mutations of the respiratory chain components in NMDs. The five complexes of the respiratory chain are schematized, and the various mutated subunits found in NMDs are reported. mtDNA-encoded subunits are highlighted in blue, while nDNA-encoded subunits are in red. Assembly factors that are not part of the mature complex are indicated in green. The structures of Complexes I–V were obtained from the Protein Data Bank (PDB) (http://www.rcsb.org/, accessed on 13 September 2021) with the accession codes PBD ID: 5LNK (Complex I), 1NEN (Complex II), 6FO0 (Complex III), 5Z62 (Complex IV), and 2XND (Complex V). For details see the text.

ATP synthase catalyzes ATP synthesis by condensing inorganic phosphate to ADP using the proton motive force generated in the mitochondrial intermembrane space by the electron transfer activity of Complexes I, III, and IV (Figure 1).

The subunits of Complexes I, III, IV, and V are encoded by nuclear and mitochodrial genes, whereas those of Complex II are only encoded by nDNA. In detail, nDNA encodes 76 proteins, while mtDNA encodes 13 proteins of the electron transport chain complexes [64,65,66].

Mutations in mtDNA or nDNA lead to the development of several clinical phenotypes, including myopathies, encephalopathies, cardiomyopathies, or complex multisystem syndromes [67]. There are more than 50 mtDNA point mutations, which lead to alterations mainly in tRNAs, also affecting rRNAs and coding genes. Deletions of mtDNA segments can also occur at the level of tRNAs or coding genes [68]. A small number of mutations occur at the nDNA level, affecting genes encoding mitochondrial proteins, i.e., subunits of the respiratory chain complexes and other mitochondrial enzymes [68,69]. Mutations in mtDNA are more frequent than those in nDNA since mtDNA is more exposed to the ROS produced by OXPHOS because of the lack of protective histones and a less efficient DNA repair system in mitochondria than that present in the nucleus [70].

Neurons and muscle fibers require an intense activity of mitochondria, from which they draw the greatest part of energy; for this reason, mtDNA mutations impairing mitochondrial functions have more serious consequences in these tissues [71]. In fact, some studies have shown that either deletions or mutations in mtDNA play an important role in the onset of aging muscle atrophy, causing a deficiency of cytochrome oxidase at the level of myofibrils and atrophy of fibers and muscles with their consequent break [72]. 

Alterations in the respiratory chain play an important role in the development of some neuromuscular diseases, pointing to deficit in energy metabolism as one of the causes of alterations in neuromuscular metabolism. Some patients with various neuromuscular diseases showed a reduced activity of two or more respiratory chain complexes as compared to the controls.

*Complex I.* Dysfunctions of mitochondrial Complex I have been linked to the development of various disorders, including neuromuscular diseases [73,74]. The most affected tissues are usually the ones with the highest energy demand, as the brain, heart, kidney, and skeletal muscle. This occurs because both structural and functional alterations of Complex I induce oxidative stress, imbalance of various metabolites, and energy depletion. Mutations in Complex I can affect its function in a mild or severe way. Several mutations in mtDNA-encoded subunits of Complex I have been associated with NMDs. Mutations have been described in all ND genes (*ND1-6*, *ND4L*) encoding the core subunits of the NADH: ubiquinone oxidoreductase complex, which are believed to belong to the minimal assembly required for catalytic functions [73]. In particular, mutations in ND subunits have also been reported in mitochondrial encephalomyopathy with lactic acidosis and stroke-like episodes (MELAS) [75], as well as in myoclonic epilepsy and ragged-red fibers (MERRF) [76]. Mutations in mtDNA affecting Complex I display different outcomes, ranging from mild functional defects [67] to a severe decrease in Complex I activity [77].

Differently from mutations found in mtDNA, those in nDNA are often associated with Complex I deficiency occurring in the neonatal period, infancy, or early childhood. Mutations in nDNA involve genes encoding for proteins forming six functional modules of Complex I, such as the N-module (NDUFV2, NDUFS1, NDUFS4, NDUFS6, NDUFA2, NDUFA6, and NDUFA12), Q-module (NDUFS2, NDUFS3, NDUFS7, and NDUFS8), ND1-module (NDUFA8, NDUFA11, and NDUFA13), ND2-module (NDUFA1, NDUFA10, and NDUFC2), ND4-module (NDUFB10 and NDUFB11), and ND5-module (NDUFB3, NDUFB8 and NDUFB9) [78,79]. Furthermore, pathological variants associated with NMDs have been found in genes encoding for accessory subunits (ACAD, FOXRED1, NDUFAF1, NDUFAF2, NDUFAF3, NDUFAF4, NDUFAF6, NDUFAF8, NUBPL, TIMMDC1, and TMEM126B) (Figure 1) [57,73].

Their clinical phenotypes frequently include Leigh syndrome (LS) [80,81], fatal infantile lactic acidosis [82], leukodystrophy with macrocephaly [83], and neonatal cardiomyopathy with lactic acidosis (NCLA) [84,85]. The neuromuscular phenotypes associated with the mutations of proteins belonging to the subunits of Complex I and its assembly factors are summarized in Table 1.

Furthermore, it is believed that mitochondrial mutations reducing Complex I activity are linked to a ROS overproduction due to increased loss of electrons. This leads to increased oxidative damages, including protein oxidation, lipid peroxidation, and DNA damage. Complex I activity is closely linked to ATP synthesis and to the maintenance of the membrane potential, thus its reduced activity causes membrane potential perturbation and a decrease in the ATP production from OXPHOS [175]. As a consequence, cells alter their metabolism by increasing the activity of cytoplasmic lactate dehydrogenase, which leads to an accumulation of lactate and consequent acidification [176].

*Complex II.* The succinate dehydrogenase complex is a heterotetramer incorporated into the inner mitochondrial membrane with the catalytic domain facing the mitochondrial matrix. It is made up of four proteins, SDHA, SDHB, SDHC, and SDHD, encoded by nuclear genes; the first two proteins form the catalytic domain, while the latter two are membrane-anchoring proteins [60,177]. 

Alterations of Complex II due to inherited homozygous mutations of its catalytic components (SDHA, SDHB, and SDHC) and of the SDHAF1 assembly factor are associated with a reduced number of neuromuscular pathologies (Table 1), including Leigh syndrome, cardiomyopathy, and infantile leukodystrophies. Such mutations can lead to a reduced functionality of this complex or to a total loss of its activity (Table 1). In addition, accumulation of succinate, increased ROS production, and reduced cell ability to synthesize ATP from oxidative phosphorylation can be observed [127]. In this context, only 2% of patients with Leigh syndrome have a deficiency of Complex II activity. Mutations generally occur at the level of the nuclear SDHA gene, causing impairments of Complex II and increased blood lactate levels. The phenotype resulting from these mutations is characterized by psychomotor retardation, muscular hypotonia, ataxia, respiratory insufficiency, and tremors [127,128].

Moreover, mutations in the gene encoding the SDHA protein have been associated with the development of rare diseases, such as familial neonatal isolated cardiomyopathy, and a late-onset neurodegenerative disease characterized by progressive optic atrophy, ataxia, and myopathy [178]. Some patients have a mutation in SDHA, which makes the enzyme unable to bind FAD, leading to a decrease in the TCA activity (the conversion rate of succinate to fumarate is decreased) and to a reduction of ATP synthesis due to decreased FADH electron transfer. Such patients show a phenotype characterized by optic atrophy, ataxia, and myopathy [79,82].

A study has found that patients with a homozygous missense mutation in the SDHA gene have reduced Complex II activity by up to 60% and 85% in skeletal and heart muscles, respectively [130], and they develop cardiomyopathy.

Heterozygous missense mutations in the SDHA gene can also lead to late-onset disease, in fact, some patients have 50% reduced activity of both Complex II and the succinate dehydrogenase enzyme. In these cases, phenotypes are characterized by progressive optic atrophy, ataxia, and myopathy [179].

*Complex III.* Neuromuscular pathologies caused by mutations affecting Complex III are not very frequent. Differently from what has been observed with diseases involving other complexes, their diagnosis is more difficult. In such cases, histological and biochemical hallmarks such as COX negative and ragged-red fibers are not present [180]. Mutations can involve genes encoding the different subunits of this complex (Table 1); in this regard, one of them is encoded by mtDNA, whereas the others are encoded by nDNA (Figure 1). In any case, all mutations cause a reduction in the activity of Complex III.

Cytochrome b (cyt b) is the only mtDNA-encoded subunit of Complex III; mutations in its gene, mitochondrially encoded cytochrome b (MT-CYB), are characterized by skeletal muscle involvement, exercise intolerance, and lactic acidosis [135]. Several studies conducted on patients’ muscle mitochondria have identified defects at the level of Complex III and cyt b in patients showing muscle weakness, progressive ataxia, lactic acidosis, and disorders of the central nervous system. Studies carried out at the molecular level have identified different types of heteroplasmic mutations. More in detail, missense and nonsense mutations have been found in the cyt b protein gene [80,136,181,182].

Other studies have reported that a 4 bp deletion in the cyt b gene results in a truncated form of cyt b associated with the development of MELAS. From a biochemical point of view, this deletion determines a reduced synthesis of iron sulfur proteins, defects in Complex III, and oxidative phosphorylation, as well as high production of hydrogen peroxide due to an increased leakage of electrons [137,183].

Most mutations affecting Complex III subunits involve nDNA genes. They encode structural proteins, such as ubiquinol-cytochrome c reductase Complex III subunit VII (UQCRQ), and assembly factors of this complex, including tetratricopeptide repeat domain-containing protein 19 (TTC19) [184], LYR motif containing 7 (LYRM7) [140], and BCS1 homolog or ubiquinol-cytochrome c reductase complex chaperone (BCS1L) [185]; their mutations cause defective Complex III assembly/stability and function.

In particular, an autosomal recessive mutation in the UQCRQ gene has been identified in some patients with severe Leigh-like syndrome, psychomotor retardation, dystonia, ataxia, and marked dementia. A reduced activity of Complex III has been found at the level of the skeletal muscle; in some cases, a reduced activity of Complex I has also been observed [144,180].

Mutations in the BCS1L gene are among the main causes of Complex III deficiency in myopathies. This gene encodes the BCS1L protein, which is an assembly factor and also a member of the ATPases associated with diverse cellular activities (AAA+). Several mutations in this gene have been identified and associated with the development of various neuromuscular diseases and other disorders, which arise from the different BCS1L expression levels in various tissues or from the effects of multiple amino acids substitutions [139]. The pathological consequences of BCS1L mutations are numerous, leading to a reduction in the activity of Complex III and sometimes of Complexes I and IV, as well as to their assembly capacity reduction of cell growth, alteration of mitochondrial morphology with subsequent mitochondrial fragmentation, and altered ROS levels and antioxidant defenses with apoptosis induction [186].

Moreover, a rare pathogenic variant in the gene encoding a catalytic subunit of Complex III, the ubiquinol-cytochrome c reductase, Rieske iron-sulfur polypeptide 1 (UQCRFS1) has recently been described [143]. Affected children had impaired Complex III assembly and activity, leading to reduced mitochondrial respiration efficiency, hypertrophic cardiomyopathy, muscular hypotonia, and weakness, together with other severe clinical multisystemic disorders [143].

*Complex IV.* Mutations affecting Complex IV can also cause neuromuscular pathologies (Table 1). This complex consists of 13 subunits, three of which are encoded by mtDNA, while the rest are encoded by nDNA. Furthermore, this is the only respiratory chain complex having different tissue-specific isoforms [162].

The three subunits encoded by mtDNA, namely MT-CO1, MT-CO2, and MT-CO3, constitute the core of the catalytic domain of Complex IV (Figure 1). Several mutations, usually heteroplasmic, can affect them, resulting in the development of various clinical phenotypes, including rhabdomyolysis, myopathy, lactic acidosis, MELAS, encephalomyopathy, and Leigh syndrome. Several mutations have been identified in MT-CO1 subunit, including microdeletions and nonsense mutations, which have been associated with the onset of motor neuron disease, multisystem mitochondrial disorders, and myopathy. Histochemical analysis of patients’ tissues has shown reduced COX II and COX III activity and inability to assemble Complex IV [145].

The missense mutation m.G6955A has been detected in the MT-CO1 gene of patients with myopathy [146]. Morphological and histochemical analyses have revealed muscles with atrophic type II fibers and subsarcolemmal accumulations of mitochondria, but not ragged-red fibers. These results are consistent with a reduced activity of Complex IV [146].

Recently, a new stop codon mutation, m.G6579A, has been identified in MT-CO1. It leads to the synthesis of a truncated form of MT-CO1 and has been associated with the development of adult-onset Leigh syndrome. Patients develop encephalopathy, brainstem and extrapyramidal signs, and have elevated blood lactate levels due to mitochondrial dysfunction. In addition, COX-deficient fibers and a reduction in the activity of Complex IV have emerged from histochemical analysis of muscle tissues [147].

Initially, only two mutations have been attributed to the gene encoding MT-CO2: a missense mutation, M153X, linked to the development of myopathy and an initiation codon mutation causing encephalomyopathy [150,151]. The latter is caused by a mutation changing the start codon from Met to Thr, leading to a dysfunctional protein or no protein production. The resulting phenotype is characterized by ataxia, optic atrophy, cognitive impairment, and muscle atrophy [150]. Subsequently, new pathogenic variants linked to MT-CO2 have been identified, determining a phenotype linked to exercise intolerance and lactic acidosis, but the most severe form identified is linked to a frame-shift mutation (m. 8042delAT) associated with the development of myopathy, bradycardia, lactic acidosis, and premature death [151,187]. In recent years, a truncated form of MT-CO2 has been identified due to the insertion of a premature stop codon (m.8088delT; p.L168X). This mutation is heteroplasmic and occurs with a high frequency in skeletal muscle, resulting in exercise intolerance with breathlessness, tachycardia, and proximal muscle weakness. Biochemical analyses of muscle tissue have highlighted a low activity of Complex IV and the absence of fibers with normal COX activity [152].

Several kinds of mutations, such as microdeletions and frameshift mutations, as well as missense and nonsense mutations, have been found in the MT-CO3 gene. In particular, a 15 bp microdeletion has been detected in the skeletal muscles of patients with ragged-red fibers suffering from myopathy and myoglobinuria and often presenting with muscle weakness, exercise intolerance, and seizures. The mutation m.G9952A results in a premature stop codon causing the loss of 13 amino acids in the COX III subunit. It has been associated with the development of MELAS and encephalopathy, implying exercise intolerance and lactic acidosis [156,162].

A novel mtDNA mutation has been associated with a deletion resulting in a frameshift and downstream stop codon in the COX III subunit. This leads to the development of rhabdomyolysis, which results in the loss of skeletal muscle protein content, in particular myoglobin. Rhabdomyolysis induced by Complex IV alterations is often associated with an increase in serum creatine kinase levels; analysis of muscle tissues has revealed a reduction in the activity of Complex IV in 45% of muscle fibers in the absence of ragged-red fibers [157].

Other important mutations can affect all those accessory proteins of Complex IV encoded by nDNA. Among these, three are associated with the onset of neuromuscular disorders, SURF1, SCO2, and SCO1. Mutations in the gene encoding SURF1, a protein useful for the assembly of Complex IV, result in pathologies such as Leigh syndrome and Charcot–Marie–Tooth disease [162].

Leigh disease causes neurodegenerative disorders with psychomotor retardation, ocular movement dysfunction, hypotonia, and metabolic acidosis. From a biochemical and molecular point of view, approximately 75% of patients have defects in the pyruvate dehydrogenase complex and in Complex IV. The latter is defective because of the absence of the SURF1 protein, which can undergo different types of mutations resulting in a lack of its production [159,160]. It has emerged that different types of mutations in the SURF1 gene, such as missense mutations, deletions. and variations of the splice sites, can lead to the onset of demyelinating Charcot–Marie–Tooth disease (CMT4). In fact, 5% of patients with this pathology have mutations in the SURF1 gene and show characteristic signs such as demyelinating neuropathy, lactic acidosis, brain magnetic resonance imaging abnormalities, and cerebellar ataxia [161].

The SCO1 gene encodes a protein involved in the assembly of COX. Three different mutations have been identified in this gene, two are associated with the development of liver disease and hypotonia while the third is associated with intrauterine developmental delay and cardiomyopathy. All mutations are associated with lactic acidosis and encephalopathy [162]. A new homozygous mutation due to a frame deletion of the gene encoding SCO1 has resulted in a new phenotype not including the symptoms listed above, but it is characterized by the onset of hypoglycemia and chronic lactic acidosis [188].

The SCO2 gene encodes a protein involved in the assembly process of Complex IV. SCO2 is a metallochaperone that picks up a monovalent copper (Cu+) ion and releases it at the level of the CuA site of Complex IV [189]. Mutations of this protein determine the onset of fatal infantile cardioencephalomyopathy, with COX deficiency in the heart and skeletal muscles. This pathology is characterized by hypotonia, hypertrophic cardiomyopathy (HCMP), and encephalopathy [163]. In general, patients with SCO2 mutations have at least one E140K missense allele and are characterized by a drastic reduction in the SCO2 protein content in the heart and skeletal muscles, along with a reduction in copper concentration in these tissues [190]. Copper deficiency has been associated with a wide range of neurological diseases, including motor neuron diseases such as Charcot–Marie–Tooth disease [164].

*Complex V.* ATP synthase is the enzymatic complex that catalyzes the last step in oxidative phosphorylation, leading to ATP synthesis (Figure 1). Mutations in the MT-ATP6 and MT-ATP8 genes, which encode subunits α and 8 of ATP synthase, respectively, can lead to NMDs (Table 1). The main mutations related to NMD development concern MT-ATP6, impairing ATP synthesis and increasing ROS production [165], but sporadic weakness has been associated with mutations found in both α and 8 subunits [172].

The mutation m.T9185C results in a leucine to proline substitution (L220P) and impairs ATP synthase function, leading to maternally inherited Leigh syndrome (MILS), neuropathy, ataxia, and retinitis pigmentosa or Charcot–Marie–Tooth disease [166]. Two other mutations, m.T8993G and m.T8993C, can cause MILS. The mutation m.T8993G determines a block of proton transfer, with a strong reduction in ATP production [165]. Two concomitant mitochondrial mutations, m.A8527G and m.C8932T, have been found in two Tunisian patients with neuromuscular disorders [167]. Such mutations are able to alter ATP synthase structure, leading to mitochondrial dysfunction.

Pathogenic mutations in Complex V subunit 8 are associated with epilepsy, sporadic weakness, infantile cardiomyopathy, hypertrophic cardiomyopathy, and ataxia [165].

## 5. Alterations of Mitochondrial Enzymes Impair Neuromuscular Functions

Many muscle pathologies are characterized by mitochondrial dysfunctions, which determine muscle degeneration through the induction of apoptosis (usually with the release of proapoptotic factors such as cytochrome c) or alterations of the mitochondrial core [191]. Typically, muscle biopsies carried out on patients with neuromuscular diseases have shown the presence of ragged-red and COX-deficient fibers, decreased activity of Complexes I and IV, and lowered ATP content [192]. Mitochondrial alterations can affect both morphology and function.

As for morphological alterations, two GTPase-dependent processes take place, mitochondrial fission and fusion. They change the shape, size, and distribution of mitochondria to meet cellular metabolic needs [40]. Alterations of mitochondrial dynamics leads to the development of various pathologies, including cancer, as well as neurodegenerative and neuromuscular disorders [193,194].

**Figure 2:**
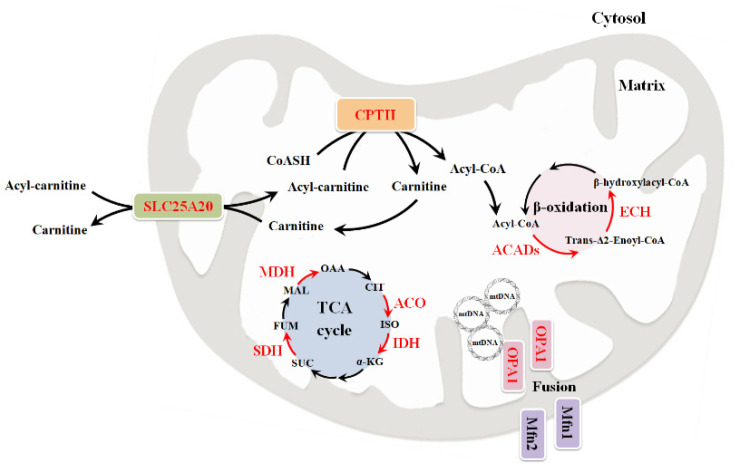
Schematic representation of altered mitochondrial enzymes in NMDs. Mitochondrial metabolic pathways involved in NMDs are shown, and mutated enzymes are highlighted in red. For details, see the text. α-KG, α-ketoglutarate; ACAD, acyl-CoA dehydrogenase; ACO, aconitase; ASP, aspartate; CIT, citrate; CoASH, coenzyme A; ECH, 2-enoyl-CoA hydratase; FUM, fumarate; IDH, isocitrate dehydrogenase; ISO, isocitrate; MAL, malate; MDH, malate dehydrogenase; Mfn1/2, mitofusins 1 and 2; OAA, oxaloacetate; OPA1, optic atrophy 1 protein; SDH, succinate dehydrogenase; SUC, succinate; TCA, tricarboxylic acid cycle.

Mitochondrial fusion needs three GTPase-dependent proteins, optic atrophy 1 protein (OPA1) and mitofusins 1 and 2 (Mfn1, Mfn2) (Figure 2) [195]. Mitofusins are involved in the fusion of external mitochondrial membranes, while OPA1 is responsible for the fusion of internal mitochondrial membranes and mtDNA mantenance [196,197,198].

Cells from patients with the OPA1 mutation have shown alterations in mitochondrial morphology, oxidative stress, and oxidative phosphorylation. These molecular alterations have been associated with mtDNA deletions and instability [199].

In some cases, patients develop a particular dominant optic atrophy phenotype, called “DOA plus”, associated with extraocular symptoms [200,201].

MFN2 dysfunctions can result from both monoallelic and biallelic pathogenic variants (Table 2). Monoallelic mutations are associated with type 2 Charcot–Marie–Tooth neuropathy (CMT2A), optic atrophy, hereditary sensory, and motor neuropathy. CMT2A is a non-demyelinating axonal peripheral neuropathy manifested by muscle weakness and atrophy that mainly affects the distal lower limbs but progressively can also affect the upper limbs [202]. Nerve biopsies from affected individuals usually show loss of large myelinated fibers and mitochondrial abnormalities but no changes in myelin. Furthermore, MFN2 mutations result in fragmentation of mitochondria and loss of their normal membrane potential [203].

Several mechanisms are involved in muscular dystrophies, inflammatory myopathies, and spinal muscular atrophy in which a reduction in the membrane potential and an altered opening of the mitochondrial permeability transition pore (mPTP) have been found. Permeability transition pores are formed by proteins that dissipate the proton concentration gradient responsible for ATP formation, thus causing its depletion [204]. Furthermore, cytochrome c emerges from these pores and induces apoptosis. The opening threshold of mPTP becomes very close to that of membrane potential; even a slight depolarization can induce its opening, leading to ATP depletion [36].

**Table 2:** Impaired mitochondrial enzymes associated with neuromuscular phenotypes. Their amino acids and nucleotide mutations are also reported. For the functions of the listed proteins see Figure 2.

Gene	Protein Name	Associated Neuromuscular Phenotypes	Pathogenic Mutations	Reference(s)
*OPA1*	Optic atrophy 1	Excercise intollerance, ataxia, and ophtalmoplegia	R455M (c.G1334A) S545R (c.C1635G) Q297X (c.C889T) A357T (c.G1069A)	[199,200]
*MFN2*	Mitofusin 2	Type 2 Charcot–Marie–Tooth neuropathy, motor neuropathy, muscle weakness, and atrophy	R95W (c.C280T) R280H (c.G839A)	[202,203]
*ACO2*	Aconitase	Truncal hypotonia, muscle atrophy, and seizures	R607C (c.1819T9 P712l (c.C2135T)	[205]
*MDH2*	Malate dehydrogenase 2	Muscle weakness, muscle atrophy, and severe hypotonia	P133L (c.C398T) P207L (c.C620T) G199Afs*10 (c596delG)	[206,207]
*CPTII*	Carnitine palmitoyl transferase II	Severe infantile hepatocardiomuscular disease and myopathy	S113L (c.S113L) P227L (c.C1196T) K414TfsX7 (c.1238_1239delAG) K642Tfsx6 (c.1926_1935DEL)	[208,209,210]
*SCAD*	Short-chain acylCoA dehydrogenase	Hypotonia, seizures, progressive myopathy, cardiomyopathy, and progressive external ophtalmoplegia	G209S (c.G625A) R171W (c.C511T)	[211]
*VLCAD*	Very-long-chain acylCoA dehydrogenase	Early onset cardiac and skeletal myopathy	F418L (c.T1372C) G401A (c.G1322A) E454K (c.G1600A) R575Q (c.G1844A)	[211]
*ETFDH*	Electron transfer flavoprotein dehydrogenase	Myopathy, dysphagia and respiratory failure, and multiple acyl-CoA dehydrogenase deficiency	A187V (c.C560T) D511N (c.G1531A)	[212,213]
*ECHS1*	Enoyl-CoA hydratase	Early onset Leigh-like syndrome, dystonia, and ataxia syndrome	A158D (c.C473A) Q159R (c.A476G) V82L (c.G244T)	[214]

*TCA cycle.* In recent decades, defects in the enzymes of this cycle have been associated with a broad spectrum of neuromuscular and neurological disorders.

The aconitase enzyme constitutes the second step of the TCA cycle and catalyzes the conversion of citrate into isocitrate (Figure 2). There are two isoforms of aconitase, cytosolic and mitochondrial. Mitochondrial aconitase is encoded by the nuclear ACO2 gene, of which several pathogenic variants have been discovered. Symptoms related to the variation of this gene are mostly related to cerebellar optic atrophy and developmental delay [69,215].

In addition, a variant of this gene has been found in a two-year-old boy with several neuromuscular symptoms, which include truncal ataxia, distal hypotonia, and impaired motor and cognitive skills. This patient has a missense mutation of the ACO2 gene causing a decrease in enzymatic activity by 80%, affecting cellular respiration and mtDNA depletion. The latter can cause defects at the level of the protein content of the electron transport chain. These data suggest that ACO2 impairment could play an important role in the regulation of some subunits of the electron transport chain [205].

Isocitrate dehydrogenase is encoded by the IDH3a gene, catalyzes the conversion of isocitrate into α-ketoglutarate (αKG), and appears to be involved in the development of neurotransmission defects (Figure 2). Altered activity of this enzyme causes a reduction in the levels of αKG, a metabolite directly involved in the synaptic transmission process. In this regard, αKG improves the activity of synaptotagmin 1, a Ca^2+^ dependent membrane protein allowing fusion between synaptic vesicles and the plasma membrane [216].

In fruit flies, loss of isocitrate dehydrogenase activity is associated with the impairment of synaptic transmission in larval neuromuscular junctions. Indeed, recent studies conducted on fruit flies have found that loss of idh3a results in reduced levels of αKG, and supplementing idh3a mutant flies with αKG could suppress synaptic transmission defects [217].

The mitochondrial malate dehydrogenase enzyme catalyzes the conversion of malate into oxaloacetate in the TCA cycle and is encoded by the MDH2 gene (Figure 2). Bi-allelic mutations of this gene have been associated with the development of severe neurological diseases that occur at an early age. A study conducted on three different patients has highlighted that malate dehydrogenase deficiency had been one of the causes leading to the onset of their childhood encephalopathy. About one year after the onset of this disease they had experienced muscle defects, including generalized muscle weakness (predominant in the lower limbs) with marked muscle atrophy, severe hypotonia, and abnormal movements [206]. Furthermore, analysis of their fibroblasts had revealed no sign of dysfunction of the respiratory chain, which could indicate the subdivision of the TCA cycle into complementary minicycles allowing a correct functioning of the respiratory chain despite the defect present in the second part of the TCA cycle [207].

*β-oxidation.* Mitochondria regulates lipid metabolism through the β-oxidation of fatty acids (Figure 2). Alterations in lipid metabolism and storage can cause cardiac and neuromuscular pathologies. In fact, a high accumulation of lipids in muscle affects mitochondrial function, which is linked to the development of myopathies [218].

Disorders associated with lipid metabolism mainly involve intracellular triglyceride degradation, carnitine uptake, or β-oxidation [219,220].

Before being degraded through the β-oxidation process, fatty acids undergo an activation step within the cytosol. Activated fatty acids are found in the form of acyl-CoA, a fatty acid bound to a molecule of coenzyme A, which is unable to cross the mitochondrial membrane. Hence, the acyl portion is transferred to a carnitine molecule, forming acyl-carnitine (Figure 2). This transfer can occur thanks to the presence of two carnitine proteins, palmitoyl transferase I (CPT I) and II (CPT II), located on the inner aspect of the outer mitochondrial membrane and in the inner mitochondrial membrane, respectively [219].

CPT II deficiency is an inherited autosomal recessive disease. Different pathogenic variants of the CPT II gene have been identified and are mainly characterized by amino acid variations or small gene deletions [221].

The most common alterations involving CPT II show a phenotype that varies according to the age at which this deficit occurs. There are three different phenotypes, a lethal neonatal form, a severe infantile hepatocardiomuscular form, and a myopathic form [208].

The first two can occur in a period ranging from birth to the first year of life, whereas the myopathic phenotype can occur from childhood to adulthood. The lethal neonatal form is characterized by liver failure, hypoketotic hypoglycemia, cardiomyopathy, respiratory distress, and/or cardiac arrhythmias. The severe infantile hepatocardiomuscular form is characterized by hypoketotic hypoglycemia, liver failure, cardiomyopathy, and peripheral myopathy [209,210]. The myopathic form is the most common disorder of lipid metabolism affecting skeletal muscle, and also the most frequent cause of hereditary myoglobinuria; it can lead to rhabdomyolysis linked to the development of myalgia and muscle stiffness [222].

The β-oxidation pathway is characterized by four intramitochondrial reactions catalyzed by the following enzymes: acyl-CoA dehydrogenase, enoyl-CoA hydratase, hydroxy acyl-CoA dehydrogenase, and ketoacyl-CoA thiolase (Figure 2). Patients with neuromuscular disorders show reduced enzymatic activity of some of them [223].

Several studies have shown that alterations of acyl-CoA dehydrogenase are associated with the onset of neuromuscular disorders. In particular, two of its three isoforms, long-chain acyl-CoA dehydrogenase (LCAD) and short-chain acyl-CoA dehydrogenase (SCAD), can undergo homozygous or heterozygous mutations in the ACADS gene and contribute to the onset of these diseases (Table 2) [224].

Deficits of very-long-chain acyl-CoA dehydrogenase (VLCAD) are related to the development of early-onset cardiac and skeletal myopathy [225,226]. Alteration of lipid metabolism determines the appearance of urinary acids, such as 3-hydroxy-dicarboxylic acids. About 60% of the mutations seen so far are associated with the mutation of a nitrogenous base from G to C in nucleotide position 1528 of the hydroxyacyl-CoA dehydrogenase (HADHA) gene [220]. Deficiencies of SCAD lead to a phenotype that includes developmental delay, hypotonia, seizures, and progressive myopathy. A variant phenotype characterized by progressive external ophthalmoplegia with ptosis, cardiomyopathy, and scoliosis has also been described. The most common variants of mutations in the gene encoding SCAD are 625 G>A and 511 C>T and lead to an excessive production of ethylmalonic acid [220].

Multiple acyl-CoA dehydrogenase deficiency (MADD) is a rare autosomal recessive disorder that may be associated with a mutation found in the gene encoding electron transfer flavoprotein (ETF) or in the gene encoding electron transfer flavoprotein dehydrogenase (ETFDH). This pathology causes metabolic alterations [212], leading to reduced ATP synthesis, high accumulation of lipids in the muscles, reduced gluconeogenesis, and neuromuscular disorders characterized by myopathic forms with proximal and axial muscle involvement and, in some cases, dysphagia or respiratory failure [212,213].

Biallelic mutations have been found in the ECHS1 gene encoding the enoyl-CoA hydratase protein, and they have been associated with early onset of a Leigh-like syndrome that is characterized by the development of progressive encephalopathy and mitochondrial deficits leading to the development of optic atrophy, developmental delay, and symptoms not related to neuromuscular disorders, such as epilepsy and bilateral hearing loss [226,227,228]. Recently, ECHS1 enzyme deficiency has been associated with the development of a new symptomatology associated with dystonia-ataxia syndrome with permanent torsional nystagmus [214]. 

## 6. Alterations of Mitochondrial Carriers and NMDs

Mitochondrial carriers (MCs) are membrane-embedded proteins localized in the inner membrane of mitochondria. They are encoded by nuclear genes belonging to a family of proteins called SLC25 or the mitochondrial carrier family (MCF), the largest solute transporter family in humans [229]. Their common function is to provide a link between the mitochondrial matrix and the cytosol by catalyzing the translocation of a large variety of solutes across the inner mitochondrial membrane. This link is indispensable for normal cell function, as many physiological processes require the participation of both intra- and extramitochondrial enzyme reactions. MCs are known to be involved in the TCA cycle, oxidative phosphorylation, fatty acid oxidation, gluconeogenesis, lipogenesis, transfer of reducing equivalents, urea synthesis, amino acid degradation, intramitochondrial DNA, RNA and protein synthesis, as well as other functions occurring between the cytosol and mitochondria. Besides such basic functions, some MCs also play an important role in regulating and balancing cytosolic and mitochondrial matrix processes, such as phosphorylation and redox potentials [230,231].

Given the central role in cell metabolism of most mitochondrial carriers, their altered function could lead to the development of several diseases mainly characterized by specific metabolic dysfunctions and depending on the biological function in intermediary metabolism [232]. In common with mitochondrial diseases resulting from mutated mitochondrial proteins, many of the MC-associated diseases frequently imply different forms of myopathy, as well as neuropathy. The neuromuscular phenotypes associated with the mutation of MFC members are reported in Table 3.

**Figure 3:**
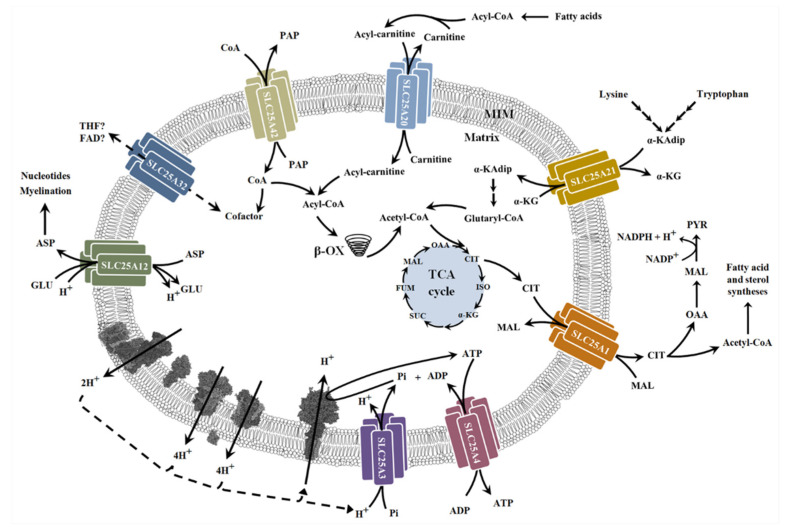
Metabolic role of the SLC25A members involved in NMDs. Mitochondrial carriers are indicated with the coding gene name, and the main transported substrates, when known, are reported. α-KAdip, α-ketoadipate; α-KG, α-ketoglutarate; ASP, aspartate; β-OX, β-oxidation; CIT, citrate; CoA, coenzyme A; FAD, flavin adenine dinucleotide; FUM, fumarate; GLU, glutamate; ISO, isocitrate; MAL, malate; MIM, mitochondrial inner membrane; OAA, oxaloacetate; PAP, adenosine 3′,5′-diphosphate; Pi, inorganic phosphate; PYR, pyruvate; SUC, succinate; TCA, tricarboxylic acid; THF, tetrahydrofolate.

*SLC25A1.* Mutations in the mitochondrial citrate carrier (CIC) have recently been associated with congenital myasthenic syndromes (CMS), a heterogeneous group of neuromuscular disorders, all characterized by an impaired neuromuscular transmission, mainly due to alterations in the neuromuscular junction [233,234,235]. CIC is encoded by the SLC25A1 gene and promotes the efflux of citrate/isocitrate from the mitochondrial matrix in exchange for cytosolic malate (Figure 3). It is now well known that CIC plays a key role in fundamental biological processes, such as fatty acid and sterol biosynthesis, gluconeogenesis, glycolysis, maintenance of chromosome integrity, and autophagy regulation [258,259].

For the first time, Chaouch A. et al. identified a homozygous missense mutation (R247Q) in the gene of the SLC25A1 protein in a pair of British siblings born to two healthy consanguineous parents (first cousins), resulting in a mild form of CMS with intellectual disability. The siblings had fatigable weakness from early childhood and abnormal jitters in several muscles associated with impaired neuromuscular junction transmission [233].

More recently, three additional unrelated CMS families with the R247Q missense SLC25A1 variant were found [236]. Clinically, these families had a similar phenotype to that of the British siblings characterized by early onset cognitive impairment and fatigable muscular weakness, which are hallmarks of impaired neuromuscular transmission [236]. Genetic analysis performed in these unrelated families revealed that patients had distinct haplotypes, proving that the R247Q SLC25A1 variant is not a founder effect, rather it is the result of a recurrent mutation associated with a relatively mild CMS phenotype implying intellectual disability. Taken together, these data suggest that SLC25A1 plays an important role in presynaptic nerve terminal activity.

Interestingly, Edvardson et al. reported that a patient having different compound heterozygous missense mutations in SLC25A1 (G130D and R282H) suffered from a neuromuscular transmission defect [260], thus confirming the existence of a correlation between SLC25A1 and congenital myasthenic syndromes. The clinical phenotype of this patient was particularly critical. She suffered from a severe neurodevelopmental disorder that included serious psychomotor retardation and hypotonia. These severe symptoms seem to highlight the important consequences of impaired citrate/isocitrate export from mitochondria. This can result in decreased TCA cycle flux, low NADPH content, increased oxidative stress, and ROS toxicity, as observed in the yeast strain expressing a protein containing both human counterpart R276H and G117D mutations. That strain exhibited a severe growth defect under stress conditions, and the recombinant mutant proteins reconstituted into liposomes exhibited a complete loss of activity [260]. Notably, this patient, unlike the others, showed prominent excretion of 2-hydroxyglutaric acid and Krebs cycle intermediates in the urine, which are features of combined D-2- and L-2-hydroxyglutaric aciduria (D/L-2-HGA), a devastating neurometabolic disorder with early lethality, previously associated with other pathogenic SLC25A1 variants [234]. In this case, the complete failure of CIC activity impairs mitochondrial metabolism and consequently cell function, resulting in a very severe phenotype.

Moreover, two novel pathogenic variants of SLC25A1 (R210X and V49M) were identified in 2020 by Li W. et al. [261]. In addition to the typical symptoms of CMS, such as occasional fatigable muscular weakness, myasthenic crisis, and epilepsy, the patient presented with abnormal metabolic signs in the urine and blood, which are proper features of D/L-2-HGA, thus demonstrating the existence of an intermediate phenotype between the two syndromes.

All this evidence suggests that the phenotypes of SLC25A1-related diseases appear to be a consecutive spectrum that include CMS, D/L-2-HGA, and the intermediate-type characterized by the combined characteristics of CMS and D/L-2-HGA. 

*SLC25A3.* Inorganic phosphate is imported into the mitochondrial matrix through phosphate carriers (PiC) for energy production and several other matrix processes. Two PiC isoforms are encoded by alternative splicing of the SLC25A3 gene [262]. PiC-A is expressed in the heart and muscle, whereas PiC-B is expressed at much lower levels in all tissues [263]. It is easy to deduce that the physiological role of PiC-A is to fulfill metabolic demands associated with contraction of muscle fibers, and that energy production deficiency is responsible for diseases involving muscle tissues. Variants of the mitochondrial phosphate carrier have been reported to be associated with cardiac and skeletal myopathies and often lactic acidosis [237,238,239].

Homozygous mutations on exon 3A affect isoform A [238], whereas other heterozygous mutations in regions outside of exon 3 affect both PiC isoforms by limiting conformational changes necessary for normal transport function [239]. Interestingly, SLC25A3 knockout mice have shown a reduced, but not completely null, mitochondrial phosphate transport activity, and consequently a lower ATP production efficiency [240]. Residual phosphate transport activity is due to other mitochondrial carriers transporting phosphate, such as the ATP-Mg^2+^/phosphate carrier isoforms [264] and dicarboxylate carrier [265], as well as uncoupling proteins UCP2, UCP5, and UCP6 [241,266]. Furthermore, when mutations involve only PiC-A, the phenotype is less severe than that observed when both PiC isoforms are mutated due to the remaining phosphate transport activity of PiC-B, albeit at a lower extent. Instead, patients with mutations in both PiC isoforms have the most severe phenotype with shorter lifespans [237].

*SLC25A4.* The SLC25A4 gene encodes AAC1, which is one of the four human mitochondrial ADP/ATP carriers. Mutations in this gene are well-recognized causes of neuromuscular diseases. AAC1 function is closely related to mitochondrial energy metabolism because of its physiological role implying the ADP transfer from the cytoplasm to the mitochondrial matrix and reciprocally the ATP transfer from the mitochondrial matrix to the cytoplasm (Figure 3). AAC1 is mainly expressed in the heart and skeletal muscle [267]; therefore, it is not surprising that AAC1 dysfunction can affect muscle energy supply [232,268].

Mutations in the SLC25A4 gene can result in either multiple mtDNA deletions or mtDNA depletion, likely due to the imbalance of mitochondrial nucleotide pools [235,242]. In both cases, mtDNA alteration causes a more or less reduced ATP production, leading to energy failure and cellular dysfunction. Thus, ATP may not only be insufficiently transferred into the cytosol but also produced in too low amounts to maintain physiological cell functions. Generally, the skeletal muscles, brain, and heart are affected as encephalo-myo-cardiomyopathy. In particular, SCL25A4 mutations manifest as adult onset progressive external ophthalmoplegia if transmitted in an autosomal-dominant way and are associated with multiple mtDNA deletions [242,243], whereas they cause a childhood onset or early adulthood onset cardiomyopathy and skeletal muscle myopathy if transmitted in an autosomal recessive way and associated with mtDNA depletion. However, there is evidence that suggests SLC25A4 mutations associated with mtDNA depletion cause the most severe phenotype and worst outcome. In fact, in depletion syndromes, congenital respiratory insufficiency occurs, resulting in impaired respiration and poor prognosis [235]. Notably, clinical phenotypes associated with different mutations have been related to the severity of transport activity impairment. In particular, mutations associated with mtDNA deletion had high residual transport activities (approximately 50%), whereas mutations associated with mtDNA depletion were completely null (<1% transport activity).

A third distinct clinical phenotype associated with two de novo mutations (A80H and R235G) found in SLC25A4 was described by Thompson K. et al. in 2018 [244]. In these cases, the mutated carrier had an intermediate residual transport activity (3–24%) compared with those of the two phenotypes associated with mtDNA deletion and depletion, being the de novo mutations in highly conserved and functionally important regions of the carrier. The reduced transport efficiency of the mutated carrier resulted in a drastic drop of energy sources, which led to mitochondrial function impairment and consequently to very severe and often fatal clinical features characterized by congenital hypotonia and profound muscle weakness requiring artificial ventilation [244].

Recently, a novel de novo heterozygous SLC25A4 variant was linked to childhood onset mild myopathy in a two-year-old girl with hypotonia and mild gross motor delays but without cardiac or eye problems or cognitive impairment [245]. The variant resulted from a substitution of the essential amino acid lysine in position 33 for the nonessential glutamine (K33Q). The SLC25A4 mutant protein overexpressed in bacteria showed a null ADP/ATP transport activity, although it was produced in normal amounts, suggesting that this mutation does not affect biogenesis, protein folding, or targeting to the mitochondrial membrane. The phenotype associated with this mutation is less severe than those of the two previous mutations; this could be explained by the fact that relative expression of the wild-type and mutant alleles and the expression of other ANT isoforms can compensate for transport defects of the mutated carrier [245].

Recently, some studies were carried out on yeast strains expressing pathogenic mutant forms of the ADP/ATP carrier isoform 2, as well as on *Caenorhabditis elegans* strain, in which the expression of ant1-1, the ortholog of human SLC25A4 [269],was reduced by RNA interference. Such investigations revealed that some molecules were able to rescue the observed phenotype, likely due to their ROS scavenger ability [270].

*SLC25A12.* The aspartate/glutamate carrier (AGC) is one of the most important MCF members playing an essential role in several metabolic processes. As AGC catalyzes an electrogenic exchange of cytosolic glutamate for mitochondrial aspartate, it is a key component of the malate–aspartate shuttle, providing mitochondria with NADH-reducing equivalents that support ATP production [271,272] (Figure 3). Two genes, SLC25A12 and SLC25A13, encode the two AGC isoforms, namely AGC1 or aralar and AGC2 or citrin, respectively. AGC1 and AGC2 are differentially expressed among tissues and during development. In particular, AGC1 is highly expressed in the adult brain, heart, and skeletal muscle, and its deficiency has been widely associated with alterations in the central nervous system and muscle, leading to the onset of more or less severe symptoms, such as developmental delay, epilepsy, hypotonia, and hypomyelination [232,246]. All mutations identified in SLC25A12 (Q590R, R252Q, and T444I) involve amino acid residues implicated in substrate binding or in the closing/opening of the carrier at the matrix side, causing transport activity impairment with a consequent reduction in ATP production. Moreover, failed mitochondrial export of aspartate, which is needed for myelin biosynthesis, compromises central nervous system functions and determines the onset of symptoms associated with defective AGC1 [273,274,275].

Recently, a mutation in the AGC1 encoding gene was found in a family of Dutch Shepherd dogs with early onset muscle weakness and inflammatory muscle biopsies. The dramatic decrease in AGC1 transport activity caused a highly oxidizing and proinflammatory muscle environment, along with inflammatory myopathy in affected dogs [247]. The primary effect of AGC1 mutation on skeletal muscles could be explained by the highly oxidative nature of canine muscle metabolism and its dependence on mitochondrial ATP. AGC1 impaired functions with the consequent inability to transfer reducing equivalents from the cytosol to mitochondria by the malate–aspartate shuttle were found to enhance oxidative stress and to support a proinflammatory milieu in the muscles from affected dogs [247].

*SLC25A20.* The mitochondrial carnitine/acylcarnitine carrier (CAC) is encoded by the SLC25A20 gene and catalyzes the exchange of cytosolic acylcarnitine for mitochondrial carnitine (Figure 3). Acylcarnitines are then oxidized in β-oxidation, representing the major source of energy for skeletal muscles and the heart [248,249]. In patients, CAC deficiency has been identified in a severe, early onset form and in a mild, infancy onset or childhood onset form ([232] and references herein). Both forms are characterized by a fasting-induced coma, muscle weakness, hypotonia, cardiomyopathy, respiratory distress, and neurological dysfunctions. SLC25A20 deficiency implies a reduction (in the mild form) or abolishment (in the severe form) of β-oxidation activity. It is likely that the accumulation of acylcarnitine and fatty acids, which cannot be oxidized, is the cause of symptoms leading to the death of patients [248,249].

*SLC25A21.* Boczonadi et al. reported a patient affected by spinal motor atrophy-like disease associated with a homozygous mutation (c.695A>G; p.K232R) in the SLC25A21 gene [250] encoding the mitochondrial ODC1 carrier [276]. It mainly transports into mitochondria 2-oxoadipate and 2-aminoadipate resulting from the degradation of lysine and tryptophan in the cytosol. The inactivation of SLC25A21 causes accumulation of 2-oxoadipate and quinolinic acid, which are toxic for neuronal cells, leading to mitochondrial deficiency, metabolic alteration, and mtDNA depletion [250].

*SLC25A32.* Neuromuscular disorders have also been associated with SLC25A32 deficiency. Member 32 of the MCF has been suggested to transport FAD and/or folate across the inner mitochondrial membrane, thus regulating mitochondrial one-carbon metabolism and redox balance [277,278]. The first two SLC25A32 mutations associated with a neuromuscular phenotype were identified in a 14-year-old patient who presented with recurrent exercise intolerance and abnormalities in the acyl-carnitine profile [251]. This condition is characteristic of multiple acyl-CoA dehydrogenase deficiency, a clinically heterogeneous disorder generally caused by defects in either ETF or ETF-ubiquinone oxidoreductase, which are proteins implicated in electron transport between the acyl-CoA dehydrogenases and the bc1 complex of the respiratory chain. Schiff et al. demonstrated that reduced mitochondrial FAD levels due to SLC25A32 impairment affected the functionality of mitochondrial respiratory chain flavoproteins, thus affecting fatty acid oxidation and oxidative phosphorylation [251]. A novel homozygous SLC25A32 mutation was identified in another patient with a more severe neuromuscular phenotype, including early onset ataxia, myoclonia, dysarthria, muscle weakness, and exercise intolerance [252]. This mutation leads to the complete absence of the functional SLC25A32 protein, justifying the more severe observed neuromuscular symptoms compared to those of the previously identified SLC25A32 variant [252]. Interestingly, oral treatment with riboflavin (the FAD precursor vitamin) raised intramitochondrial FAD levels, leading to an improvement in the clinical condition of the patient [252].

*SLC25A42.* Only one mitochondrial coenzyme A (CoA) transporter is known, encoded by the SLC25A42 gene. It mediates the transport of CoA and, less efficiently, of dephospho-CoA (dPCoA) into mitochondria in exchange for intramitochondrial adenosine 3′,5′-diphosphate (PAP) and (deoxy)adenine nucleotides. CoA plays a critical role in several metabolic pathways, such as the Krebs and urea cycles, β-oxidation, heme biosynthesis, and branched-chain amino acid oxidation [279]. Recently, one SLC25A42 variant was found in different patients with variable clinical features attributable to mitochondrial myopathy. Some affected individuals also presented with developmental delay, lactic acidosis, and encephalopathy [253,254,280]. Recently, pathological variants of SLC25A42 were found in six patients with encephalomyopathy [255]. They showed heterogeneous clinical symptoms from mild to severe motor and speech impairment. Metabolic acidosis, dystonia, and lactic acidosis were also observed. Furthermore, in all patients mitochondrial dysfunction was revealed [255].

Considering the fundamental physiological role of this carrier in energy metabolism, it is not surprising that intramitochondrial deficiency of CoA, due to its defective transporter, frequently gives rise to disorders involving highly energy demanding tissues, such as nerves and muscles [281]. Supporting evidence was obtained using one SLC25A42-deficient zebrafish model, in which a severe phenotype with muscle disorganization and weakness was observed. These features could be rescued by expressing the wild-type human SLC25A42 gene, but not by employing the mutant variant, confirming the close association between SLC25A42 mutation and mitochondrial myopathy in humans [253].

*SLC25A46.* Various mutations found in the SLC25A46 gene were identified and related to several mitochondrial neurodegenerative and neuromuscular diseases, including inherited optic atrophy, Charcot–Marie–Tooth type 2, Leigh syndrome, progressive myoclonic ataxia, and lethal congenital pontocerebellar hypoplasia. SLC25A46 mutations result in a varied spectrum of clinical presentations, ranging from premature death to mild manifestations in adulthood [282,283]. Van Dijk et al. identified SLC25A46 loss-of-function mutations in patients from a Dutch family with a particular subtype of lethal congenital pontocerebellar hypoplasia (PCH1) characterized by the combination of spinal muscular atrophy and PCH. In this study [256], the authors provided strong evidence that loss of functional SLC25A46 could be the cause of motor neuron degeneration, suggesting a novel classification for SLC25A46-associated PCH1 as PCH1D in order to distinguish it from other PCH1 subtypes characterized by mutations in other genes involved in RNA metabolism and gene expression [256].

To date, SLC25A46 transport function is not known. Rather, this protein seems to be involved in mitochondrial dynamics and cristae remodeling, similar to its closest homolog Ugo1, a yeast protein involved in mitochondrial fusion. Moreover, unlike other MCs, SLC25A46 localizes to the outer mitochondrial membrane [284]. It was demonstrated that knockdown of this protein in human cultured cells compromised mitochondrial fission process, leading to mitochondrial hyperfusion, alterations in the endoplasmic reticulum morphology, impaired cellular respiration, and premature cellular senescence [284,285].

In recent years, Drosophila models of neuromuscular diseases were developed to increase the understanding on their pathological mechanisms. In *D. melanogaster*, two genes were identified as human SLC25A46 homologs, CG8931 and CG5755, encoding dSLC25A46a and dSLC25A46b, respectively. Their physiological role is not yet known, but they seem to be involved in mitochondrial fission and dynamics as the human homolog [257]. In agreement with the phenotypes observed in patients with SLC25A46 deficiency, dSLC25A46a and dSLC25A46b knockdown Drosophila models displayed locomotor impairment and defects in neuromuscular junctions compromising synaptic function. Moreover, severe structural and functional mitochondrial defects associated with ROS accumulation and ATP level reductions were also observed in dSLC25A46a and dSLC25A46b mutant fruit flies [257,286].

## 7. Diagnosis and Treatments

Neuromuscular disorders lead to the development of a very wide and diversified symptomatology, thus making accurate diagnosis difficult. Clinical manifestations are raised by a large number of genetic, proteic, and metabolic alterations, making it difficult to develop an effective treatment. In fact, there is no single therapy aimed at treating NMDs, but a series of treatments are carried out, the purpose of which is to improve the patients’ quality of life by reducing disease progression rather than by rescuing defects. More attention is given to personalized therapies for individual patients in order to develop a therapeutic path that considers the patient’s clinical history and symptoms [7].

In order to diagnose NMDs, a series of tests is conducted with the aim of simplifying and carrying out a more correct and rapid identification of the pathology, allowing a discrimination between the different subtypes of NMDs. For example, electromyography is able to measure muscle electrical activity and to assess the presence of damage to the peripheral nervous system and muscles as well as in suspected cases of myopathies, myositis, myasthenia, and other severe forms of NMDs. Since many NMDs are hereditary, genetic molecular testing is extremely important to predict their inheritance [287].

The information reported in this review shows how the diagnosis of NMDs can be facilitated if the analysis of clinical phenotypes is sustained by investigations of nuclear genes, encoding mitochondrial proteins, and mtDNA. Hence, biochemical analysis could highlight mitochondrial dysfunctions caused by impairment of mitochondrial proteins. Despite the absence of definitive treatments for NMDs, today different types of interventions can be carried out to improve the patients’ quality of life, slowing down or blocking disease development. The most common treatments include drug therapy, surgery, and, more recently, genetic therapies [6]. Since most NMDs are associated with mitochondrial dysfunction, many drug therapies target mitochondria. The goals of these treatments are to increase the activity of the electron transport chain acting as ROS scavengers or to modulate mitochondrial biogenesis [288].

Administration of antioxidants, cofactors, and vitamins should be useful to increase respiratory chain activity. In this regard, patients’ diets can be supplemented with coenzyme Q_10_ (CoQ10), idebenone, or riboflavin (vitamin B2) to improve the flow of electrons through the transport chain, bypassing defective mitochondrial complexes [289]. Randomized clinical trials investigated the effects of therapeutic supplementation with CoQ10 in patients with MELAS [290], showing no statistically significant differences when compared to placebo. Similar results were observed in a study using idebenone, a synthetic analog of CoQ10 with a shorter chain [291].

Antioxidants acting as ROS scavengers have been tested to decrease ROS overproduction that is due to a malfunction of the electron transport chain and leads to cell damage and death observed in many NMDs. Hence, the administered molecules can regenerate glutathione, the main antioxidant compound whose activity can be reduced in some forms of NMDs. Molecules endowed with this property are vitamin E or its analogue Epi-743, which is more stable and better tolerated. This substance has been used in patients with Leigh syndrome [292]. In several studies involving patients with Leigh syndrome, treatment with EPI-743 was associated with reversal of disease progression [293] and clinical improvement [294]. Mitochonic acid-5 (MA-5) was reported to increase cellular ATP level and to reduce mitochondrial ROS production in mitomice, i.e., a mouse model that mimics the typical phenotypes of human mitochondrial disease [295], as well as fibroblasts of patients with mitochondrial diseases, such as MELAS, Leigh Syndrome, and DOA [296]. Furthermore, MA-5 was supposed to protect IBM patients’ fibroblasts from death by inducing the expression of OPA1 and DRP1 genes, thus improving the imbalance of mitochondrial fusion/fission processes [297].

Diet therapy can be used to treat some patients affected by NMDs. In the case of AGC1 deficiency, a ketogenic diet was adopted [298]. Dehlin et al. administrated a ketogenic diet with carbohydrate restriction and increased fat/protein ratio (from 1:1 to 4:1) [298]. The patient exhibited psychomotor improvement along with resumed myelination. Such clinical improvements could be due to the decreased NADH/NAD^+^ ratio achieved by carbohydrate restriction. Furthermore, the consequent increase in cytosolic aspartate by malate dehydrogenase could enhance myelin lipid biosynthesis with a beneficial effect on neuromuscular symptoms. L-carnitine was also administrated as a dietary supplement to patients with mitochondrial myopathy [299]. In some clinical trials, oral L-carnitine supplementation promoted a significant increase in exercise tolerance and oxygen consumption [300,301].

Finally, some molecules modulate mitochondrial biogenesis, acting on the transcriptional coactivator peroxisome proliferator-activated receptor γ coactivator-1α (PGC-1α), whose upregulation increases the biosynthesis and number of mitochondria within the cell. PGC-1α can be activated by deacetylation carried out by sirtuin 1 (Sirt1), by phosphorylation carried out by AMP- activated protein kinase (AMPK), or by PPAR γ that upregulates its gene expression [288].

Recently, therapies using antisense oligonucleotides (ASOs) and gene replacement therapies have been developed. ASOs are small, modified fragments of nucleic acids that are synthesized in such a way as to be complementary to an RNA sequence that must be degraded or modified by splicing [302]. This strategy is applied for the treatment of several NMDs [303]. 

Gene replacement therapies are based on replacing the defective genes that cause NMDs [304]. In order to develop a gene therapy, it is necessary to know the mutated gene responsible for the disease and to replace it with the therapeutic gene. Common therapeutic strategies include gene replacement, gene knockdown, gene repair, and disease-modifying gene therapy [305]. Gene transfer is performed using viral and nonviral vectors. In particular, the most used vector is the adeno-associated virus (AAV). The administration of this therapy is carried out intravenously. Recently, an AAV-based gene therapy study was carried out on a mouse model of Complex I deficiency Leigh syndrome, knocking out the NUDFS4 gene. The administration of AAV2/9-CMV-NUDFS4 was able to rescue completely the activity of Complex I in the heart and skeletal muscle [306]. An AAV9-based vector was used to rescue of skeletal muscle phenotypes, such as exercise intolerance in the NUDFS3 knockout mouse model. After treatment with AAV9-CMV-NUDFS3, the mice showed normal Complex I activity and improved motor coordination and muscle strength [307]. A different adeno-associated virus vector was used to test gene therapy for SLC25A46 deficiency in a mouse model. AAV-PHP-SLC25A46 was able to improve the phenotype of the mouse model, improving coordination and body weight in a dose-dependent manner [308].

To date, few therapeutic strategies are used for the treatment of NMDs, and many of them aim to slow the disease rather than cure it. Furthermore, there is no evidence of an approved gene therapy or ASO aimed at restoring the functionality of mitochondrial proteins. Up-to-date details on conducted or ongoing clinical trials are reported in the recent reviews of Liufu et al. [309], Almannai et al. [254], Tinker et al. [310], and Pitceathly et al. [311]. Many innovative therapeutic treatments are being developed in order to have targeted therapies for each type of NMD that take into account the history and progression of the disease.

## 8. Conclusions

Neuromuscular diseases exhibit specific features, including impaired muscle and nervous functions, the causes of which are not yet fully known.

It is known that mitochondrial metabolism defects are implicated in NMDs. A large body of evidence indicates that mitochondrial metabolism plays a key role in muscle and peripheral nerve dysfunctions, affecting the progression of NMDs. Alterations in some mitochondrial processes, including the TCA cycle (involving ACO2 and MDH2), OXPHOS, as well as β-oxidation (involving CPTII, LCDA, and ECHS1) imply defects in energy supply, leading to impaired muscle and moto neurons functions. Notably, the loss of mitochondrial functions also affects signal transmission in the nervous system as well as the synthesis and release of neurotransmitters.

In recent decades, the role of several MCF members has emerged in a wide range of diseases, including NMDs. Alterations in the translocation of molecules from mitochondria to the cytosol, and vice versa, can affect cell functions, leading to the impairment of neuromuscular functions, as found for mutations in the SLC25A1, SLC25A3, SLC25A12, and SLC25A42 genes.

Understanding the link between impaired mitochondrial metabolism and NMDs is essential to identify new biomarkers and tools for advancing early diagnosis. Furthermore, such a new insight should be useful for developing new therapeutic strategies and drugs able to improve the patients’ quality of life.

As mitochondrial functions are impaired in several neuromuscular diseases, mitochondrial therapies could be new promising future therapeutic targets. In the light of the latest knowledge, gene therapy represents a new interesting tool for treating NMDs. Currently, there is no evidence of gene therapy targeting nuclear genes encoding enzymes involved in mitochondrial metabolic pathways. Despite this, rescue of mutations in genes causing NMDs should be developed in the future.

In conclusion, this review highlights the role of several mitochondrial proteins involved in the complex etiology of NMDs, showing that further research is still required to fully understand the link between mitochondrial disfunctions and neuromuscular metabolism in order to identify useful targets for new possible therapies. 

## Figures and Tables

**Table 1:** Impaired mitochondrial respiratory complex subunits associated with neuromuscular phenotypes. Their amino acids and nucleotide mutations are also reported.

Gene/Protein	Function	Associated Neuromuscular Phenotype(s)	Pathogenic Mutant(s)	Reference(s)
*Complex I Subunits*				
*MTDN1*	H^+^ translocation	MELAS, dystonia, spasticity, and myopathy	T164A (m.A3796G) G131S (m.3697A) E214K (m.G3946A) Y215H (m.T3949C)	[86,87]
*MTDN3*	H^+^ translocation	Leigh syndrome	S34P (m.T10158C)	[88]
*MTDN4*	Putative proton channel	MELAS	T109A (m.A11084G)	[89]
*MTDN5*	Putative proton channel	MELAS	E145G (m.A12770G) M237L (m:A13045C)	[90]
*MTDN6*	H^+^ translocation	MELAS	A74V (m.G14453A)	[91]
*NDUFA1*	Assembly/stability	Leigh syndrome, hypotonia, nystagmus, and decreasedreflexes	G8R (c.G22C) R37S (c.G215C)	[92]
*NDUFA2*	Assembly/stability	Leigh syndrome, hypertrophic cardiomyopathy, and developmental delay	Exon 2 Skipping (c.G208A+5)	[93]
*NDUFA6*	Assembly/stability	Intrauterine growth retardation, respiratory insufficiency, and lactic acidosis	C115Y (c.G344A)	[82]
*NDUFA8*	Assembly/stability	Severe neonatal hypotonia, dysmorphic features, and epilepsy	E190K (c.G325A) A224V (c.C671T)	[84]
*NDUFA10*	Assembly/stability	Leigh syndrome and delayed psychomotor development	M1? (c.A1G) Q42R (c.A425G)	[94]
*NDUFA11*	Assembly/stability	Hypertrophic cardiomyopathy and no motor development	Exon 2 skipping (IVS1_5 GtoA)	[95]
*NDUFA12*	Assembly/stability	Leigh syndrome, progressive loss of motor abilities, scoliosis, and dystonia	R60X (c.C178T)	[96]
*NDUFA13*	Assembly/stability	Delayed development, hypotonia, abnormal eye movements, and poor feeding	R57H (c.G170A)	[97]
*NDUFB3*	Assembly/stability	Myopathy, hypotonia, developmental delay, and lactic acidosis	G70X (c.G208T) W20R (c.T64C)	[79,98]
*NDUFB8*	Assembly/stability	Leigh syndrome, respiratory failure, seizures, hypotonia, cardiac hypertrophy, failure to thrive, and severely delayed psychomotor development	P76Q (c.C277A) C144W (c.C432G)	[99]
*NDUFB9*	Assembly/stability	Progressive hypotonia	R47L (c.G140T) L64P (c.T191C)	[100]
*NDUFB10*	Assembly/stability	Cardiomyopathy and lactic acidosis	E70X (c.206_207insT) C107S (c.T319C)	[101]
*NDUFB11*	Assembly/stability	Hypertrophic cardiomyopathy and lactic acidosis	W85X (c.G254A) S96P (c.C286T) Y108X (c.T320G) P110S (c.C328T)	[102,103]
*NDUFC2*	Assembly/stability	Leigh syndrome	H58L (c.A173T) c.346_*7del	[104]
*NDUFS1*	NADH oxidation	Growth retardation, axial hypotonia, and dystonia	R241W (c.C829T) D252G (c.A863G) M707V (c.T2227G)	[105]
*NDUFS2*	NADH oxidation	Neonatal lactic acidosis and hypertrophic cardiomyopathy	R228Q (c.G283A) P229Q (c.C686A) S413P (c.T1237C)	[106]
*NDUFS3*	NADH oxidation	Leigh syndrome, severe axial dystonia with oral and pharyngeal motor dysfunction, dysphagia, and tetraparetic syndrome	T145I (c.C434T) R199W (c.C595T)	[80]
*NDUFS4*	Assembly/stability	Muscular hypotonia, absence of visual and auditive attention, and cardiac defects	T96X (c.delG289) R106X (c.C316T)	[107]
*NDUFS6*	Assembly/stability	Fatal infantile lactic acidosis	C115Y (c.G344A)	[82]
*NDUFS7*	NADH oxidation	Leigh syndrome, feeding problems, dysarthria, and ataxia	V122M (c.G384A)	[108]
*NDUFS8*	NADH oxidation	Leigh syndrome, poor feeding, and episodes of apnea and cyanosis	P79L (c.C236T) R102H (c.G305A)	[109]
*NDUFV2*	NADH oxidation	Hypertrophic cardiomyopathy and truncal hypotonia	Exon 2 skipping (c.IVS2+5_+8del)	[110]
*Complex I Assembly Proteins*				
*ACAD9*	Assembly	Myalgia, hypotonia, hypertrophic cardiomyopathy, and severe lactic acidosis	L98S (c.T223A) A220V (c.C659T) R414C (c.C1240T) R532T (c.C1594T)	[111,112,113,114,115]
*FOXRED1*	Assembly	Leigh syndrome, congenital lactic acidosis, athetoid movements of the limbs in early childhood, and hypotonia	Q323X (c.C694T) N430S (c.A1289G)	[116]
*NUBPL*	Assembly	Developmental delay, short stature, myopathy, nystagmus, and ataxia	G56R (c.G166A) L104P (c.T311C) F242L (c.C726G)	[117]
*NDUFAF1*	Assembly	Hypertrophic cardiomyopathy, developmental delay, lactic acidosis, and hypotonia	T207P (c.A1001C) K253R (c.A1140G)	[118]
*NDUFAF2*	Assembly	Ataxia, lethargy, nystagmus, and hypotonia	R45X (c.C182T)	[119]
*NDUFAF3*	Assembly	Axial hypotonia, no eye contact, and wide anterior fontanel	G77R (c.G229C) R122P (c.G365C)	[120]
*NDUFAF4*	Assembly	Cardiomyopathy	L65P (c.T194C)	[120,121]
*NDUFAF6*	Assembly	Focal seizures, decreased movement and strength, ataxia, lactic acidosis, and Leigh syndrome	Q99R (c.A296G)	[122]
*NDUFAF8*	Assembly	Leigh syndrome	F18SfsX32 (C.45_52Ddup) F55L (c.C165G)	[123]
*TIMMDC1*	Assembly	Infantile-onset hypotonia, delayed or minimal psychomotor development, dysmetria, dyskinetic movements, nystagmus, and Leigh syndrome	R255X (c.C673T)	[124]
*TMEM126B*	Assembly	Exercise intolerance, muscle weakness, myalgia, and hypertrophic cardiomyopathy	Q70X (c.C208T) D133N (c.G397A) G212V (c.G635T)	[125,126]
*Complex II Subunits*				
*SDHA*	Succinate oxidation	Leigh syndrome and neonatal dilated cardiomyopathy	G355E (c.G1700A) A524V (c.C1607T)	[127,128,129,130]
*SDHAF1*	Assembly factor	Spastic quadriplegia and psychomotor regression	R55P (c.G164C) G355E (c.G1700A)	[131]
*Complex III Subunits*				
*MTCYB*	Catalytic subunit	Exercise intolerance, encephalomyopathy, and cardiomyopathy	G166X (m.G15242A) G166E (m.G15243A) G251C (m.G15498A) G290D (m.G15615A) L13fsX50 (m.1478del4)	[132,133,134,135,136,137]
*BCS1L*	Assembly factor	Muscle weakness, Leigh syndrome, and myopathy	T50A (c.A148G) D103IfsX8 (c.A306T) E133DfsX23 (c.399delA)	[136,138,139]
*LYRM7*	Chaperon protein	Progressive weakness, severe psychomotor regression, generalized hypotonia, inability to walk, and severe spastic tetraparesis	D25N (c.G73A)	[140]
*TTC19*	Assembly factor	Ataxia and spastic paraparesis	Q173X (c.C517T) L219X (c.T656G) Q277X (c.C829T)	[131,141]
*UQCC3*	Assembly factor	Hypotonia, delayed development, and lactic acidosis	V20E (c.T59A)	[142]
*UQCRFS1*	Catalytic subunit	Cardiomyopathy	V14D(c.T41A) R204X (c.C610T) V72_81del10 (c.G215-1C)	[143]
*UQCRQ*	Binds and stabilization of cytochrome c	Leigh-like syndrome, severe psychomotor retardation, dystomia, and ataxia	S45F (c.C208T)	[144]
*Complex IV Subunits*				
*MT-CO1*	Reduction of O_2_ to H_2_O	MELAS, myopathy, myoglobinuria, motor neuron disease, exercise intolerance, epilepsy, and Leigh syndrome	G226X (m.G6578A) Q232K (m.C6597A) K265fs271X (m.A6698del) G351D (m.G6955A)	[145,146,147,148,149]
*MT-CO2*	Acceptor of electrons from cyt c	Encephalomyopathy, myopathy, and hypertrophic cardiomyopathy	M1T (m.T7587C) K29M (m.T7671A) M153X (m.8042delAT) L168X (m.8088delT)	[150,151,152,153]
*MT-CO3*	Putative oxygen uptake regulator	Myopathy, muscle weakness, exercise intollerance, and seizures	W58X (m.G9379A) V91A (m.T9478C) P118QfsX124 (m.9559del) W248X (m.G9952A)	[154,155,156,157]
*SURF-1*	Assembly factor	Leigh syndrome, Charcot–Marie–Tooth disease	P119L (c.C356T) N178fsX8 (c.531_534del) R192W (c.C574T) Q251X (c.C751T)	[158,159,160,161]
*SCO-1*	Assembly factor	Hypotonia and cardiomyopathy	G124E (c.C385G) Q251X (c.C751T)	[162]
*SCO-2*	Metallochaperone	Fatal infantile cardioencephalomyopathy, hypotonia, HCMP, and Charcot–Marie–Tooth disease	Q53X (c.158T) E140K (c.G418A) P169T (c.C505A) S225F (c.C674T)	[163,164]
*Complex V Subunits*				
*MT-ATP6*	Participation in the unidirectional H^+^ transfer	MILS, ataxia, and Charcot–Marie–Tooth disease	I24T (m.T8597C) P136S (m.C8932T)	[165,166,167,168,169,170]
*MT-ATP8*	Assembly/stability	Epilepsy, tetralogy of Fallot, weakness, infantile cardiomyopathy, hypertrophic cardiomyopathy, and ataxia	P39L (m.C8481T) W55R (m.T8528C) W55X (m.G8529A)	[168,169,170,171,172,173,174]

MELAS: mitochondrial encephalomyopathy with lactic acidosis and stroke-like episodes; MILS: maternally inherited Leigh syndrome; HCMP: hypertrophic cardiomyopathy; IVS: intervening sequence.

**Table 3:** Impaired mitochondrial carrier proteins associated with neuromuscular phenotypes. The amino acids and nucleotides mutations are also reported. For the functions of the listed proteins see Figure 3.

Gene	Protein Name	Associated Neuromuscular Phenotype(s)	Pathogenic Mutation(s)	Reference(s)
*SLC25A1*	Citrate carrier (CIC)	Congenital myastenic syndrome	R247Q (c.G740A) G130D (c.G389G) R282H (c.G845G) R210X (c.C628C) V49M (c.G145GA)	[233,234,235,236]
*SCL25A3*	Phosphate carrier (PIC)	Cardiac and skeletal myopathy	c.158-9A>G G72E (c.G215A) L200W (c.T599G) G296_S300delinsQIP (c.886-898delins7)	[237,238,239,240]
*SLC25A4*	ADP/ATP carrier isoform 1 (AAC1)	Adult onset progressive external ophtalmoplegia, adult onset cardiomyopathy, skeletal myopathy, and childhood onset mild myopathy	A80H (c.G239A) R235G (c.C703G) K33Q (c.A97T) A123D (c.C368A)	[231,241,242,243,244,245]
*SLC25A12*	Aspartate/glutamate carrier isoform 1 (AGC1)	Hypotonia, epilepsy, hypomyelination, muscle weakness, and inflammatory myopathy	R252Q (c.G1058A) Q590R (c.A1769G) T444I (c.C1331T)	[246,247]
*SLC25A20*	Carnitine/acylcarnitine carrier (CAC)	Muscle weakness, hypotonia, cardiomyopathy, and respiratory distress	Q238R (c.A713G) R275Q (c.G824A)	[248,249]
*SLC25A21*	2-Oxodicarboxylate carrier (ODC1)	Spinal motor atrophy-like disease	K232R (c.A695G)	[250]
*SLC25A32*	Folate transporter (MFT)	Excercise intolerance, early onset ataxia, myoclonic dysarthria, and muscle weakness	c.-264_31delins14 W142X (c.G425A) R147L (c.G440A)	[251,252]
*SLC25A42*	Coenzyme A transporter	Developmental delay, encephalomyopathy, mild and severe motor impairment, and myopathy	N291D (c.A871G) c.380 +2T>A F173_175RFS (c.522_524del)	[253,254,255]
*SLC25A46*	SLC25A46	Type 2 Charcot–Marie–Tooth neuropathy, Leigh syndrome, and progressive myoclonic ataxia	T142I (c.C425T) R246X (c.A736T) L348P (c.T1022C) R340C (c.C1018T) E335D (c.A1005T) L138R (c.T413G)	[256,257]

## Abbreviations

Ach	acetylcholine
ALS	amyotrophic lateral sclerosis
ASOs	antisense oligonucleotides
BCS1L	ubiquinol-cytochrome c reductase complex chaperone
CMS	congenital myasthenic syndrome
CMT	Charcot–Marie–Tooth disease
DMPK	myotonic dystrophy protein kinase
DOA	dominant optic atrophy FSHD, facioscapulohumeral muscular dystrophy
HCMP	hypertrophic cardiomyopathy
IBM	inclusion body myositis
LS	Leigh syndrome
MADD	multiple acyl-CoA dehydrogenase deficiency
MD	muscular dystrophy
MELAS	mitochondrial encephalomyopathy with lactic acidosis and stroke-like episodes
MERRF	myoclonic epilepsy and ragged-red fibers
MILS	maternally inherited Leigh syndrome
NCLA	neonatal cardiomyopathy with lactic acidosis
NMD	neuromuscular disease NMJ, neuromuscular junction
PCH	pontocerebellar hypoplasia
SCAD	short-chain acyl-CoA dehydrogenase
SDH	succinate dehydrogenase
SMN	survivor motor neuron
